# Radiomics Analysis and Correlation With Metabolic Parameters in Nasopharyngeal Carcinoma Based on PET/MR Imaging

**DOI:** 10.3389/fonc.2020.01619

**Published:** 2020-09-08

**Authors:** Qi Feng, Jiangtao Liang, Luoyu Wang, Jialing Niu, Xiuhong Ge, Peipei Pang, Zhongxiang Ding

**Affiliations:** ^1^Department of Radiology, Affiliated Hangzhou First People's Hospital, Zhejiang University School of Medicine, Hangzhou, China; ^2^Hangzhou Universal Medical Imaging Diagnostic Center, Hangzhou, China; ^3^Institutes of Psychological Sciences, Hangzhou Normal University, Hangzhou, China; ^4^Zhejiang Chinese Medical University, Hangzhou, China; ^5^GE Healthcare Life Sciences, Hangzhou, China; ^6^Translational Medicine Research Center, Key Laboratory of Clinical Cancer Pharmacology and Toxicology Research of Zhejiang Province, Affiliated Hangzhou First People's Hospital, Zhejiang University School of Medicine, Hangzhou, China

**Keywords:** nasopharyngeal carcinoma, positron emission tomography, magnetic resonance imaging, radiomics, staging

## Abstract

**Objective:** Accurate staging is of great importance in treatment selection for patients with nasopharyngeal carcinoma (NPC). The aims of this study were to construct radiomic models of NPC staging based on positron emission tomography (PET) and magnetic resonance (MR) images and to investigate the correlation between metabolic parameters and radiomic features.

**Methods:** A total of 100 consecutive cases of NPC (70 in training and 30 in the testing cohort) with undifferentiated carcinoma confirmed pathologically were recruited. Metabolic parameters of the local lesions of NPC were measured. A total of 396 radiomic features based on PET and MRI images were calculated [including histogram, Haralick, shape factor, gray level co-occurrence matrix (GLCM), and run length matrix (RLM)] and selected [using maximum relevance and minimum redundancy (mRMR) and least shrinkage and selection operator (LASSO)], respectively. The logistic regression models were established according to these features. Finally, the relationship between the metabolic parameters and radiomic features was analyzed.

**Results:** We selected the nine most relevant radiomic features (six from MR images and three from PET images) from local NPC lesions. In the PET model, the area under the receiver operating characteristic (ROC) curve (AUC), accuracy, sensitivity, and the specificity of the training group were 0.84, 0.75, 0.90, and 0.69, respectively. In the MR model, those metrics were 0.85, 0.83, 0.75, and 0.86, respectively. Pearson's correlation analysis showed that the metabolic parameters had different degrees of correlation with the selected radiomic features.

**Conclusion:** The PET and MR radiomic models were helpful in the diagnosis of NPC staging. There were correlations between the metabolic parameters and radiomic features of primary NPC based on PET/MR. In the future, PET/MR-based radiomic models, with further improvement and validation, can be a more useful and economical tool for predicting local invasion and distant metastasis of NPC.

## Introduction

Nasopharyngeal carcinoma (NPC) is a special tumor of the head and neck which is the main characteristic disease in South Asia (1). It is of great importance to appropriately predict the disease stage because proper therapy strategies are based on the current stage. The preferred treatment for early NPC is radiotherapy. However, locally advanced or advanced NPC patients should be treated with a combination of radiation and chemotherapy. Radiomics models based on ^18^F-fluorodeoxyglucose positron emission tomography (FDG-PET) and magnetic resonance imaging (MRI) could provide additional useful information for NPC staging (2).

The integrated synchronization of ^18^F-FDG PET/MR can simultaneously provide the morphological information of MRI and the molecular metabolic information of PET imaging through a single scan and realize the accurate fusion of MRI anatomical imaging and PET functional imaging. Chan et al. (3) conducted both whole-body PET/MR and PET/CT examinations on 113 patients with pathologically confirmed NPC. The study showed that, for tumor staging of NPC, PET/MR improved the accuracy of head and neck tumor detection and could better show the mapping tumor extension, especially the intracranial invasion, than PET/CT. Cheng et al. (4) performed PET/CT-MRI scans on 35 patients with NPC. The study indicated that PET/MR was more efficient in characterization and visualization and showed high lesion detection and good image quality of NPC compared with PET/CT. Some studies have shown that the combination of PET and MRI images and the comprehensive analysis of the molecular metabolism and microstructure characteristics of the tumors are of great value in the differential diagnosis and prognosis analysis of tumors (5, 6). However, there are relatively few PET/MR studies on the staging of NPC. In this study, ^18^F-FDG PET/MR was used to determine the early and late stages of NPC.

In theory, the metabolic imaging of PET can quantitatively and early reflect the heterogeneity of tumor. The preferred semi-quantitative parameter for primary and metastatic NPC in PET is standardized uptake value (SUV). Since SUV_max_ only reflects the highest tumor volume of the ^18^F-FDG perturbation value, the intake and overall metabolic of area of interest (ROI) were not assessed. Larson et al. (7) introduced the metabolic tumor volume (MTV) and total lesion glycolysis (TLG) for the assessment of important parameters.

Radiomics is a rapidly developing new technique for disease diagnosis and auxiliary detection (8). Tumor heterogeneity is a recognized cancer feature in biology, and visualization of tumor heterogeneity plays a key role in evaluating tumor invasiveness. The study of the heterogeneity of cancer foci by radiomic analysis has become a hot topic in the field of medical imaging of cancer. Radiomics provides a promising method in the diagnosis and prediction of many cancers, such as glioblastoma (9), lung cancer (10), prostate cancer (11), breast cancer (12), and colorectal cancer (13, 14). Moreover, Zhang et al. (15) conducted a multi-parameter MRI radiomic study on 118 advanced NPC patients and found that the selected radiomic features had different degrees of correlation with the T stage, N stage, and clinical stage. Du et al. (16) performed PET/CT examination on 76 patients with NPC. The study showed that machine learning methods in radiomics can distinguish local recurrence vs. inflammation. However, there is still a lack of PET/MR-based radiomic studies on NPC.

In this study, we will construct radiomic models based on ^18^F-FDG PET/MR for NPC staging and investigate the correlations between the metabolic parameters and radiomic features.

## Materials and Methods

### Patients

In this study, patients with pretreatment NPC (all pathologically non-keratinized undifferentiated carcinoma) who were examined at the Hangzhou Universal Medical Imaging Diagnostic Center from June 2017 to October 2019 were collected; all patients underwent PET/MR examination before treatment. Before the examination, all patients signed an informed consent. This study was approved by the local ethics committee (no. KT2018024), and all methods were implemented in accordance with the Declaration of Helsinki.

All patients were staged according to the 8th edition of the American Joint Committee on Cancer (AJCC)/Union for International Cancer Control (UICC) TNM staging system (17). The inclusion criteria are as follows: NPC patients with pathologically confirmed non-keratinized undifferentiated carcinoma; nasopharyngeal lesions were found for the first time without any treatment such as chemotherapy or radiotherapy; clear pretreatment PET/MR images of the whole body and head and neck can be obtained; and PET/MR examination was performed between 40 and 60 min after injection of the imaging agent. The exclusion criteria are as follows: patients who had received any form of treatment (such as radiotherapy, chemotherapy, etc.) before PET/MR examination; patients with a history of other head and neck malignancies or other systemic malignancies; PET or MRI images do not meet the diagnostic criteria (such as metal or motion artifacts); patients with MRI contraindication or intolerance; and SUV value suspected to have deviation (such as high blood sugar or low radiation purity of the FDG drug). A total of 100 consecutive NPCs who met the criteria were included. NPC patients were divided into early group (stages I and II) and advanced group (stages III and IV) according to the TNM staging system.

### PET/MR Imaging Protocol

^18^F-FDG PET/MR scans were performed using GE integrated TOF PET/MR (GE SIGNA, Wisconsin, USA). The patients fasted for more than 6 h and drank clear water. Strenuous exercise was prohibited before the injection of ^18^F-FDG. Blood glucose was controlled below 7.8 mmol/L. The patients were injected with ^18^F-FDG at a dose of 3.7 MBq/kg and underwent whole-body PET/MR examination after urination. PET images were collected and reconstructed using 3D mode, time-lapse technique, and point spread function during whole-body MRI examination. A local PET/MR scan of the head and neck, from the base of the skull to the supraclavicular bones, was then performed. Finally, whole-body and local PET, MRI, and PET/MR fusion images were obtained.

### Radiomics Analysis

**Figure 1:**
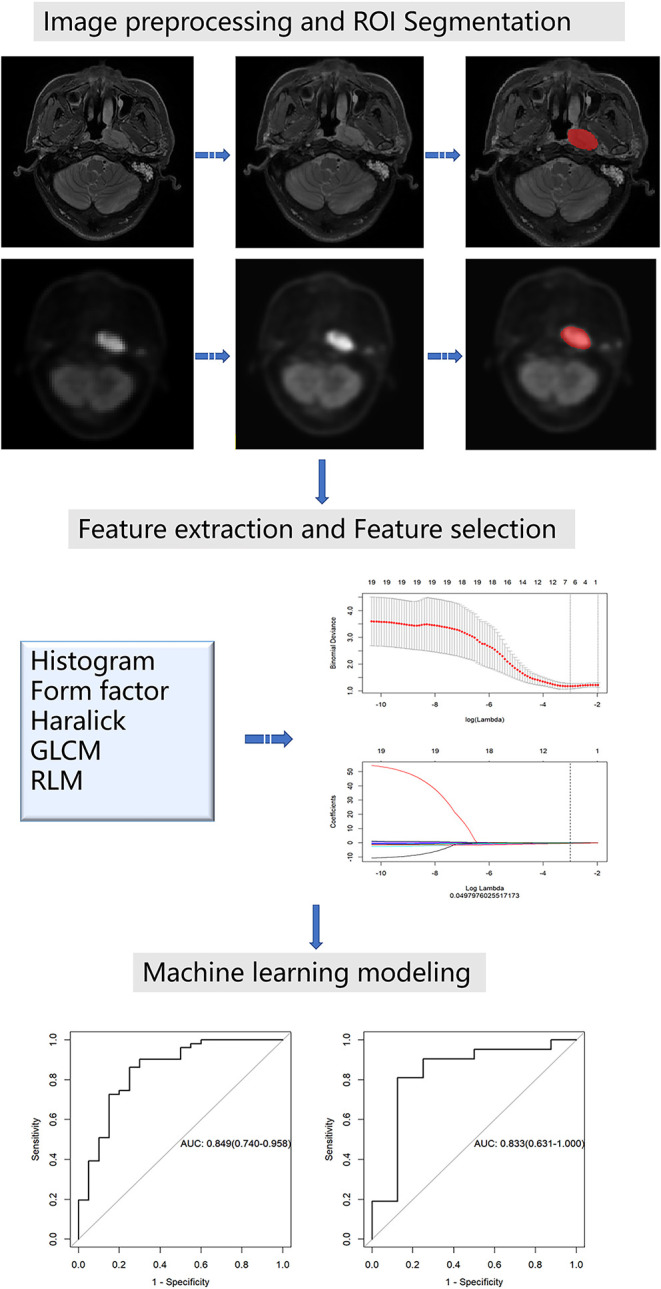
Workflow of radiomics analysis for NPC staging.

Image preprocessing was conducted using the Artificial Intelligence Kit (A.K) software which was developed by GE Healthcare. The A.K software has been registered and approved. It realizes several key steps of radiomics and has already been applied to some radiomics studies, including ourselves (18, 19). The image resolution was adjusted to 1 mm × 1 mm × 1 mm for resampling. The image was transformed into the same layer thickness through the linear difference value, i.e., 1 mm layer thickness. Then, image gray unified adjustment to 0–255 was done for standardization. The maximum value of grayscale is 255 and the minimum value is 0; the rest were converted linearly. An example before and after the preprocessing of images is shown in Figure 1.

For ROI segmentation, the T2-weighted images (T2WI) from the local head and neck scan and the corresponding PET images were imported into ITK-SNAP software (version 2.2.0; www.itksnap.org). On the T2WI, the edges of the primary NPC were manually delineated layer by layer, excluding the normal tissues and posterior pharyngeal lymph nodes that were not invaded. The segmentation boundaries of the PET images and T2WI coincide. All segmentations were conducted by a neuroradiologist with 12 years of work experience. Finally, the segmentation results of the T2WI and the PET images were derived.

For feature extraction, firstly, all the unsegmented raw data of the T2WI and PET images were imported into the A.K software, and then the corresponding ROI data were imported in batches. The selection parameters include histogram, Haralick, shape factor, gray level co-occurrence matrix (GLCM), and run length matrix (RLM) with steps 1, 4, and 7. Finally, radiomic features were extracted in batches from all the data.

For feature selection, the extracted radiomic feature tables of the T2WI and PET images were imported into the A.K software for feature selection. Then, we divided the data in a ratio of 7:3, i.e., 70% of the training set and 30% of the testing set. The outliers in the table were replaced with the average values and the data was standardized. Feature selection was conducted on the two groups of data, respectively (the total feature number for both PET and MR was 396). We used two feature selection methods: maximum relevance and minimum redundancy (mRMR) and least shrinkage and selection operator (LASSO). Firstly, mRMR was used to eliminate redundant and irrelevant features. Next, we chose the LASSO regression model, which is suitable for the dimension reduction of high-dimensional data to select the predictive radiomic features of the training data. In order to avoid overfitting, 10-fold cross-validation with minimum criteria was used. These two-dimensional reduction methods have been well-used in some radiomics studies (16, 20).

In machine learning modeling, according to the selected features, the logistic regression models of T2WI and PET were constructed using machine learning methods. The model's performance in the training and testing groups was assessed using receiver operating characteristic (ROC) curves and accuracy.

Figure 1 shows the workflow of the radiomics analysis for NPC staging.

### Measurement of PET Metabolic Parameters

Various metabolic parameters were measured using the PET VCAR software in a GE Healthcare AW 4.6 post-processing workstation by a neuroradiologist with 12 years of work experience. The PET/MR image sequences of the local head and neck scans were opened. The adaptive threshold method was used to determine the uptake boundary of the primary lesion (21), which determined 40% of the SUV_max_ in ROI as the tumor boundary. The ROI recognition box size was adjusted, and the high uptake areas such as normal tissues and metastatic lymph nodes were excluded from the ROI range in combination with the MRI structure image. Finally, three metabolic parameters of ROI, namely, MTV, SUV_max_, and TLG, were recorded.

### Statistical Analysis

Statistical analyses for clinical data comparison were performed using SPSS (version 22.0, IBM). Data of continuous variables conforming to normal distribution were expressed as the mean ± standard deviation. Chi-square (χ^2^) test was used for the comparison of counting data, *t*-test for the comparison of measurement data, and Pearson's analysis was used for the correlation between the metabolic parameters and radiomic features, which were normally distributed. All statistical methods of the radiomics analysis process were conducted with the A.K software and R software (version 3.5.2; http://www.Rproject.org).

## Results

### Comparison of Clinical Data

Table 1 shows the results of statistical analysis of the demographics and clinical data. There were no statistically significant differences in age, gender, the metabolic parameters (SUV_max_, MTV, and TLG) and clinical stage between the training group and the testing group (*P* > 0.05).

**Table 1:** Clinical data comparison of NPC patients in the training and the testing groups.

	**Training group**	**Testing group**	**Statistic**	***p-*value**
Sample size	70	30	NA	NA
Age (years, mean ± SD)	52.23 ± 12.33	50.40 ± 13.68	−0.658	0.512
Gender (male/female)	56/14	22/8	0.544*	0.461*
SUV_max_	10.91 ± 4.76	10.03 ± 3.78	−0.899	0.371
MTV	9.87 ± 7.38	11.31 ± 9.29	0.824	0.412
TLG	51.06 ± 46.95	57.61 ± 41.46	0.661	0.510
Clinical staging (I/II/III/IV)	5/14/38/13	2/6/19/3	1.250*	0.767*

Statistics were analyzed with t-test, unless otherwise indicated. SD, standard deviation; SUV_max_, maximum standard unit value; MTV, metabolic tumor volume; TLG, total lesion glycolysis; NA, not applicable. P-value, 0.05. *χ^2^ test was performed.

### Radiomics Analysis Results

**Figure 2:**
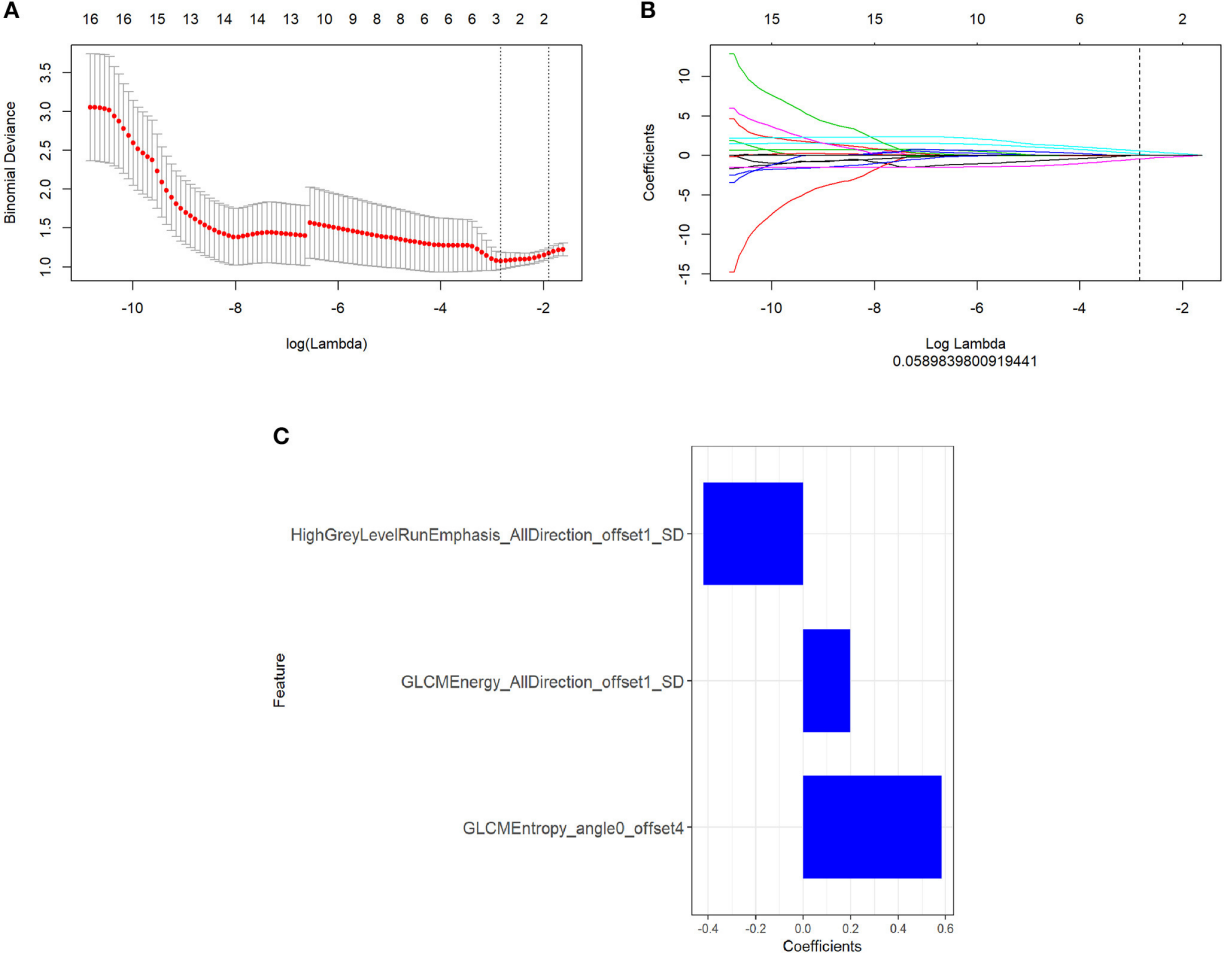
**(A)** The error rate curve. **(B)** LASSO coefficient λ graph. Coefficient λ was selected in the LASSO using a 10-fold cross-validation. We chose the coefficient λ with the lowest error rate. **(C)** The remaining features of the positron emission tomography (PET) images after feature selection.

**Figure 3:**
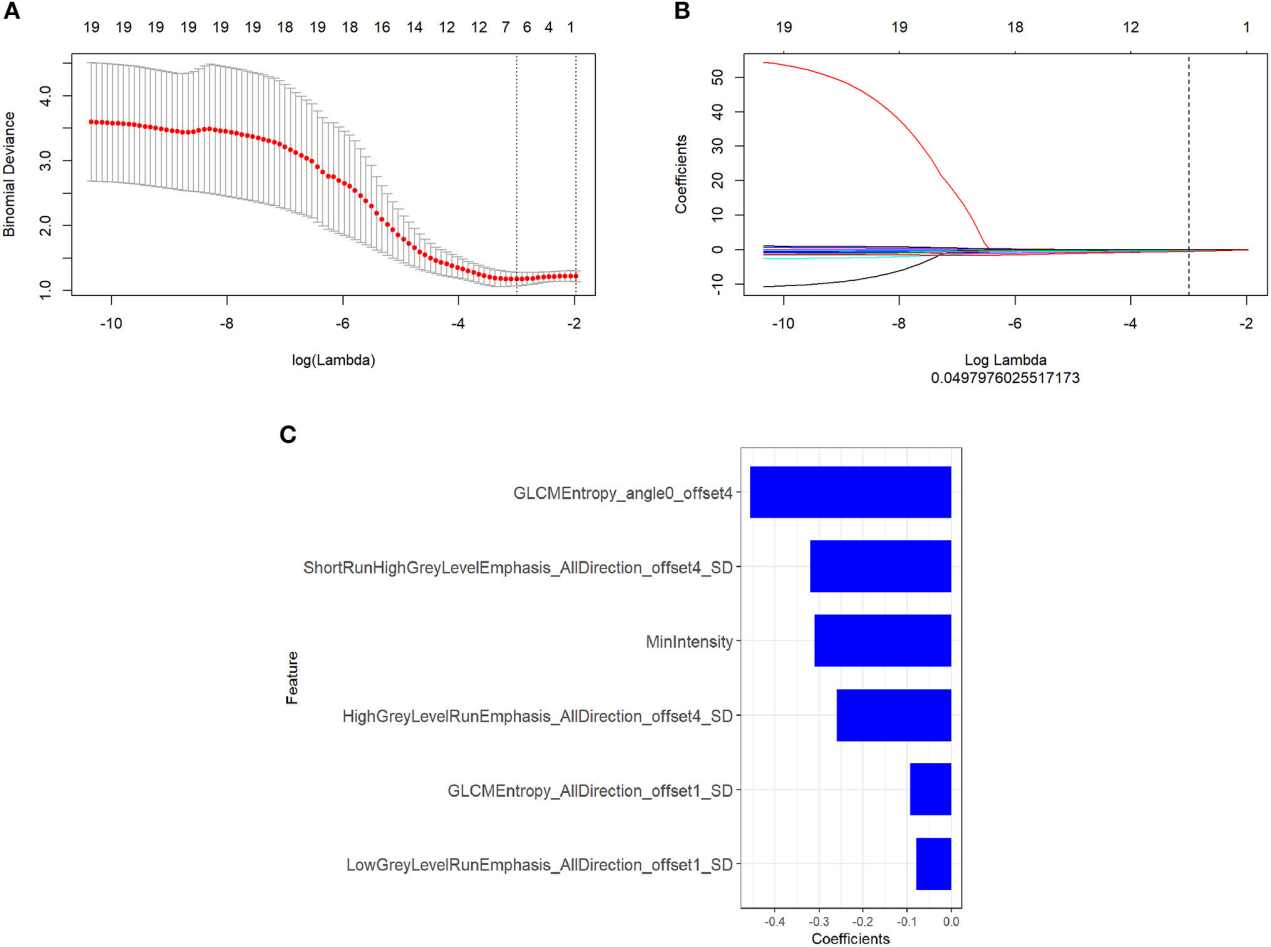
**(A)** The error rate curve. **(B)** LASSO coefficient λ graph. We chose the coefficient λ with the lowest error rate. **(C)** The remaining features of the magnetic resonance (MR) images after feature selection.

There were 396 features calculated for the PET and MR data. For the PET data, after mRMR, the remaining feature number was 20. After LASSO, three features were retained (Figures 2A–C). For the MR data, after mRMR and LASSO, the remaining feature numbers were 20 and 6, respectively (Figures 3A–C). The type and formula of the selected features are shown in Table 2.

**Table 2:** Type and formula of the selected features in positron emission tomography (PET) and magnetic resonance (MR) data.

**Feature**	**Type**	**Formula**
PET_GLCMEnergy_AllDirection_offset1_SD	GLCM	$\underset{i,j}{\sum }g{\left(i,j\right)}^{2}$
PET_GLCMEntropy_angle0_offset4	GLCM	$-\underset{i,j}{\sum }g\left(i,j\right)\log_{2}\left(i,j\right)$
PET_HighGreyLevelRunEmphasis_AllDirection_offset1_SD	RLM	$HGRE\left(\theta \right)=\frac{1}{n_{r}}\overset{N}{\underset{j=i}{\sum }}\overset{M}{\underset{i=1}{\sum }}p\left(i,j,\theta \right)i^{2}$
MR_LowGreyLevelRunEmphasis_AllDirection_offset1_SD	RLM	$LGRE\left(\theta \right)=\frac{1}{n_{r}}\overset{N}{\underset{j=i}{\sum }}\overset{M}{\underset{i=1}{\sum }}\frac{p\left(i,j,\theta \right)}{i^{2}}$
MR_ShortRunHighGreyLevelEmphasis_AllDirection_offset4_SD	RLM	$SRHGE\left(\theta \right)=\frac{1}{n_{r}}\overset{N}{\underset{j=i}{\sum }}\overset{M}{\underset{i=1}{\sum }}\frac{p\left(i,j,\theta \right)i^{2}}{j^{2}}$
MR_GLCMEntropy_AllDirection_offset1_SD	GLCM	$-\underset{i,j}{\sum }g\left(i,j\right)\log_{2}\left(i,j\right)$
MR_MinIntensity	Histogram	Minimum intensity value
MR_HighGreyLevelRunEmphasis_AllDirection_offset4_SD	RLM	$HGRE\left(\theta \right)=\frac{1}{n_{r}}\overset{N}{\underset{j=i}{\sum }}\overset{M}{\underset{i=1}{\sum }}p\left(i,j,\theta \right)i^{2}$
MR_GLCMEntropy_angle0_offset4	GLCM	$-\underset{i,j}{\sum }g\left(i,j\right)\log_{2}\left(i,j\right)$

*In the formulas, g is a gray level co-occurrence matrix (GLCM), where i, j are the spatial coordinates of g(i,j). For the GLCM parameters, i is a gray level, j is a gray value, and N is the number of classes of gray levels. For the run length matrix (RLM) parameters, n_r_ is the number of runs, N is the number of classes of gray levels, and M is the size in voxels of the largest region found*.

**Figure 4:**
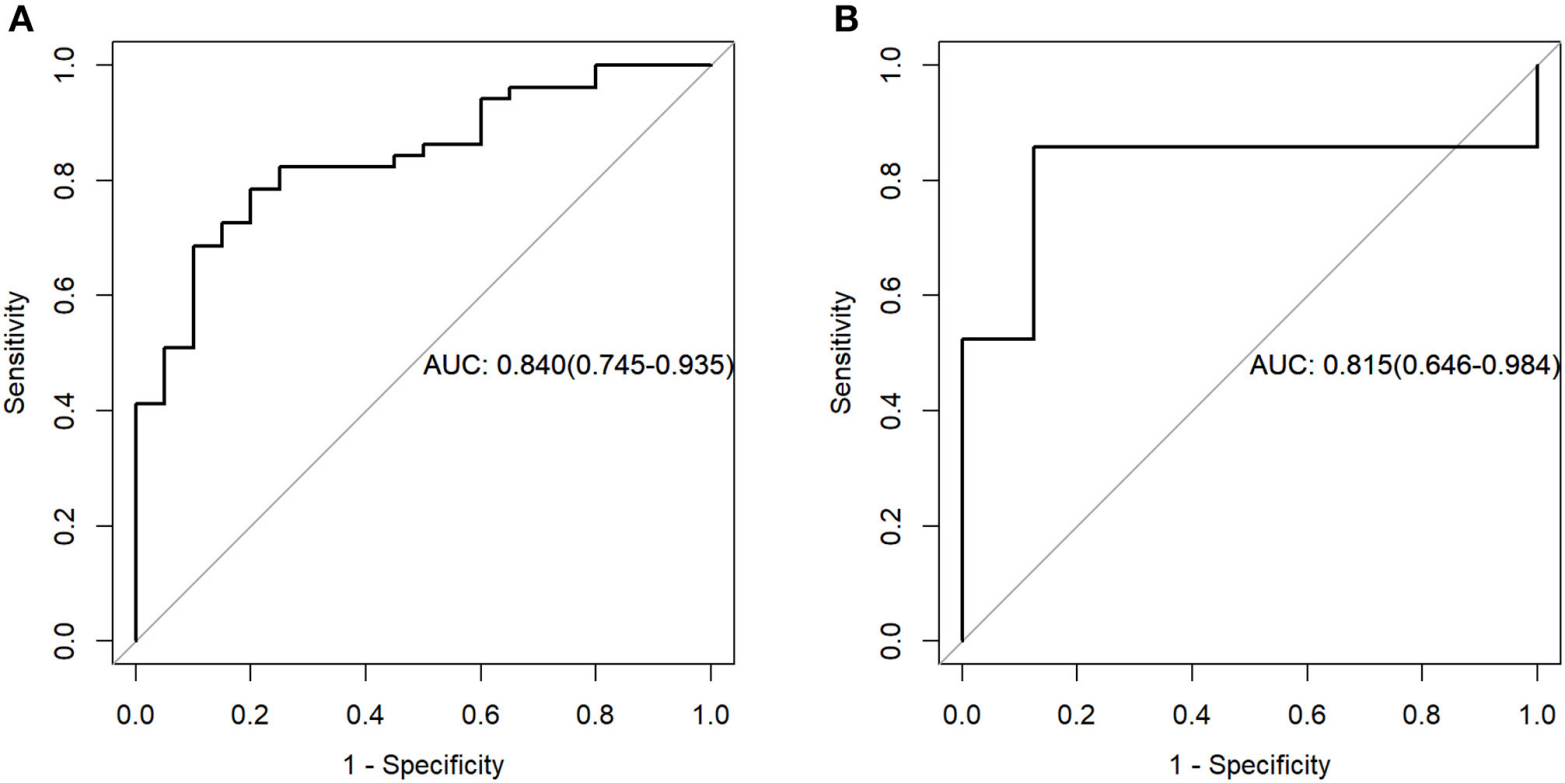
Receiver operating characteristic (ROC) curves of the training set **(A)** and testing set **(B)** in the positron emission tomography (PET) data.

For the PET data, according to the three selected features, the logistic regression algorithm was used to construct the classification model of the training group and the testing group. The area under the ROC curve (AUC), accuracy, sensitivity, and the specificity of the training group were 0.84, 0.75, 0.90, and 0.69, respectively. The corresponding indexes of the testing group were 0.82, 0.86, 0.88, and 0.86, respectively (Figure 4). The AUC values were very close in the two groups, and the fitting degree of the model was considered to be good. The cutoff values of radscore for training group and testing group were 1.01 and 0.74, respectively.

**Figure 5:**
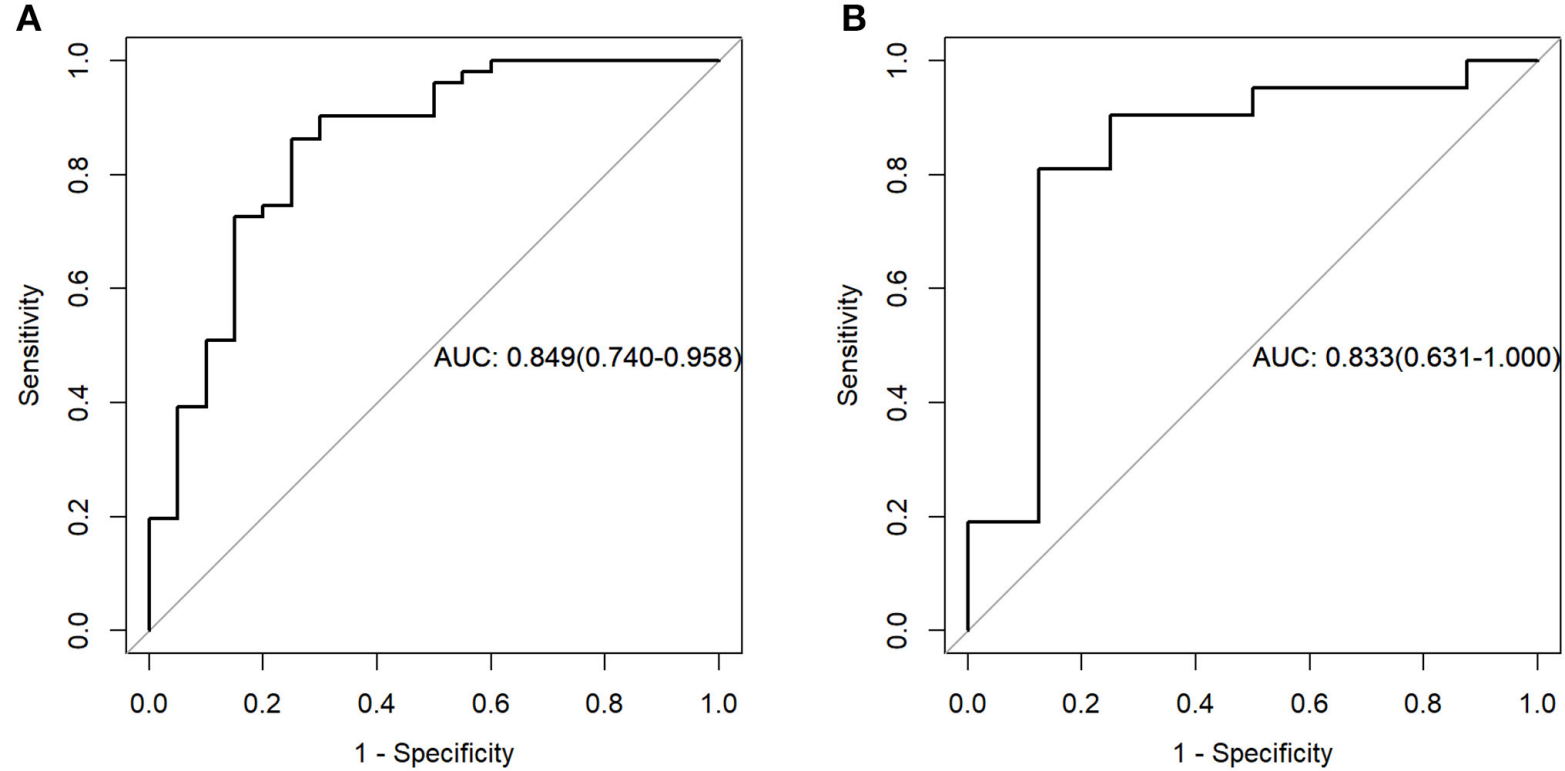
Receiver operating characteristic (ROC) curves of the training set **(A)** and testing set **(B)** in the magnetic resonance (MR) data.

**Figure 6:**
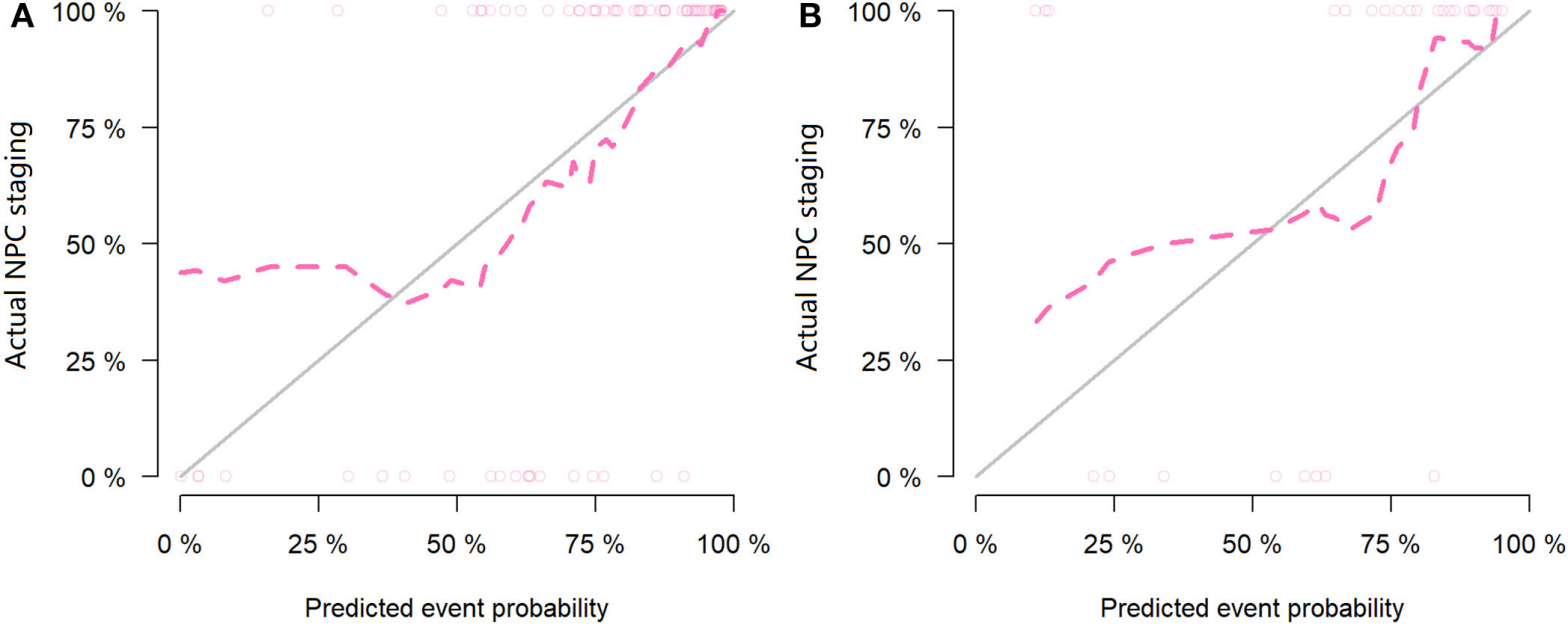
Calibration curves of the training set **(A)** and testing set **(B)** in the positron emission tomography (PET) data. The *red line* is the fitting line and represents the actual value corresponding to the predicted value.

**Figure 7:**
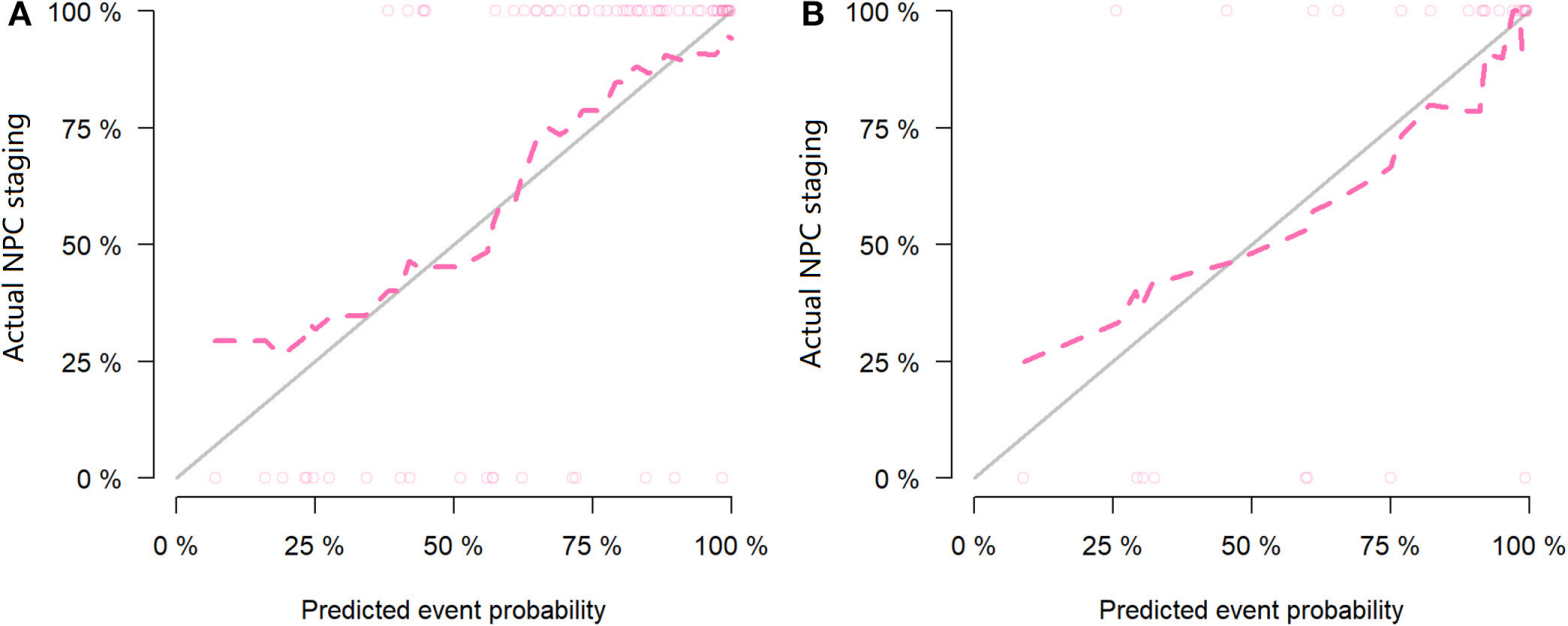
Calibration curves of the training set **(A)** and testing set **(B)** in the magnetic resonance (MR) data. The *red line* is the fitting line and represents the actual value corresponding to the predicted value.

For the MR data, logistic regression algorithm was used to construct the classification model of the training group and the testing group according to the six selected features. The AUC, accuracy, sensitivity, and specificity of the training group were 0.85, 0.83, 0.75, and 0.86, respectively. The corresponding indexes of the testing group were 0.83, 0.83, 0.88, and 0.81, respectively (Figure 5). The fitting degree of the model was also considered to be good. The cutoff values of the radscore for the training and testing groups were 0.64 and 0.89, respectively. The radscore formula is shown in Supplementary Data. Calibration curves of the PET and MR data are shown in Figures 6, 7.

### Correlation Between the Radiomic Features and PET Metabolic Parameters

In the PET model, Pearson's correlation analysis showed that the feature (GLCMEntropy_angle0_offset4) was significantly positively correlated with the MTV and TLG (*R* = 0.70 and 0.73, *P* < 0.01). The feature (HighGreyLevelRunEmphasis_AllDirection_offset1_SD) were negatively correlated with TLG (*R* = −0.33, *P* < 0.01). More correlation coefficients are shown in Table 3.

**Table 3:** Correlations between the radiomic features and PET metabolic parameters in the PET model.

**Feature**	**SUV_**max**_**	**MTV**	**TLG**
GLCMEnergy_AllDirection_offset1_SD	−0.12	−0.18	−0.20*
GLCMEntropy_angle0_offset4	0.20	0.70**	0.73**
HighGreyLevelRunEmphasis_AllDirection_offset1_SD	−0.21*	−0.29**	−0.33**

The values in the table are the correlation coefficients. SUV_max_, maximum standard unit value; MTV, metabolic tumor volume; TLG, total lesion glycolysis. *P < 0.05; **P < 0.01.

In the T2WI model, Pearson's correlation analysis showed that the three features (MinIntensity, GLCMEntropy_angle0_offset4, and HighGreyLevelRunEmphasis_AllDirection_offset4_SD) were negatively correlated with MTV (*R* = −0.45, −0.45, and −0.30, respectively, *P* < 0.01) and TLG (*R* = −0.47, −0.50, and −0.37, respectively, *P* < 0.01). More correlation coefficients are shown in Table 4.

**Table 4:** Correlations between the radiomic features and PET metabolic parameters in the MR model.

**Feature**	**SUV_**max**_**	**MTV**	**TLG**
LowGreyLevelRunEmphasis_AllDirection_offset1_SD	−0.13	−0.05	−0.09
ShortRunHighGreyLevelEmphasis_AllDirection_offset4_SD	0.01	−0.16	−0.15
GLCMEntropy_AllDirection_offset1_SD	0.11	−0.09	−0.06
MinIntensity	−0.15	−0.45**	−0.47**
HighGreyLevelRunEmphasis_AllDirection_offset4_SD	−0.26**	−0.30**	−0.37**
GLCMEntropy_angle0_offset4	−0.14	−0.45**	−0.50**

The values in the table are the correlation coefficients. SUV_max_, maximum standard unit value; MTV, metabolic tumor volume; TLG, total lesion glycolysis. **P < 0.01.

## Discussion

We selected the nine most relevant radiomic features (six from MR images and three from PET images) from local NPC lesions. The correlations between the radiomic features and the SUV_max_, MTV, and TLG metabolic parameters were discussed. The clinical value of the radiomic model in evaluating the NPC stage was also analyzed. The results showed that the constructed PET and MR radiomic models had high diagnostic performance for NPC staging, and there was a certain correlation between the metabolic parameters and some radiomic features.

Burri et al. (21) found that 40% of the SUV_max_ based on PET as the boundary of the lesion had the best correlation with the pathological and physiological characteristics of the tumor, so this study used this method to measure the metabolic parameters. In addition, we also combined the anatomical information provided by the MRI structure to exclude some interfering factors. There are several metabolic parameters representing tumor functional information, but the most representative, the SUV_max_, MTV, and TLG, parameters were included in this study (22). The metabolic parameters of primary NPC represent the clinical parameters of tumor function, but the uptake of ^18^F-FDG cannot always accurately reflect the physiological state of the tumor (23).

In recent years, more and more evidences show that the analysis of radiomics of medical images can better reflect the potential spatial variation and heterogeneity of the tumor endosomal intensity, which will generate more prediction and prognostic information (13, 24). Du et al. (16) used machine learning methods to analyze post-therapy NPC PET/CT images and found that, compared with conventional indicators, radiomics signatures showed higher AUC values (0.867–0.892 vs. 0.817) in the differentiation between local recurrence and inflammation. Zhuo et al. (25) studied the multi-modality MR images of 658 patients with non-metastatic NPC. It was found that the radiomic features based on MRI could divide NPCs into subtypes with different survival modes, which showed better performance than the TNM staging system. Zhang et al. (15) performed radiomics nomogram combined with multi-parametric MRI-based radiomic features with the TNM staging system. It showed improved prognostic ability in advanced NPC over the TNM staging system. But these studies have not included PET images. We used the T2WI and PET imaging features based on the local lesions of NPC to evaluate its application value in NPC staging. In this study, the AUC values of the T2WI and PET models were 0.85 and 0.84 in the training group and 0.83 and 0.82 in the testing group, respectively, showing good diagnostic efficacy for NPC staging.

Among the nine radiomic features extracted from the PET/MR images that were highly correlated with NPC stage, four were GLCM features, four were RLM features, and one was a histogram feature. The feature “MinIntensity” was the histogram parameter, which represents the minimum intensity of the 3D image matrix. GLCM describes texture by studying the spatial correlation characteristics of the grayscale. The advantage is that the spatial relationship of the distance and angle between two pixels can be considered simultaneously. The “GLCM_Energy” value ranges from zero to one. Constant image energy is one. The value is higher when the image has good homogeneity or the pixel is very similar. The value of “GLCM_Entropy” represents the complexity of the symbiotic matrix, and the larger the value, the more complex is the symbiotic matrix. RLM is used to obtain the length matrix by calculating the probability of the continuous occurrence of pixels in different directions and steps to describe the complexity of the lesion, the degree of change, and the texture thickness.

Theoretically, the uptake capacity of the tumor to ^18^F-FDG can quantitatively quantify tumor heterogeneity at an early stage, while the radiomic features based on PET images can provide more comprehensive details, which are attributed to pathological factors such as tumor proliferation, angiogenesis, tumor necrosis, and hypoxia (26). Therefore, it is suggested that there should be some intrinsic relationship between the metabolic parameters representing tumor uptake capacity and the radiomic features representing tumor heterogeneity. Our study showed that the metabolic parameters had different degrees of correlation with the selected radiomic features. The feature “GLCMEntropy_angle0_offset4” of the PET images had the strongest positive correlation with the metabolic parameters MTV and TLG, indicating that the more complex the symbiotic matrix of tumor is, the larger the uptake volume and the amount of glycolysis are. However, the correlations between the other radiomic features and metabolic parameters were relatively low. Some studies also found that there was a certain correlation between the radiomic features and PET metabolic parameters. A study on non-small-cell lung cancer based on PET/CT found that some texture features like volume of the lesion were highly positively correlated with MTV, the CT average density was moderately positively correlated with SUV, and CT kurtosis was moderately positively correlated with MTV (27). However, another PET/CT study on non-small-cell lung cancer showed that texture and shape features had stronger correlations with MTV and GTV compared to SUV measurements (28). The results of our study are consistent with the second study, in this respect. The uptake process of ^18^F-FDG is the potential expression of biological processes, and the measured MTV and TLG can indirectly reflect tumor proliferation, angiogenesis, tumor necrosis, etc. (29), which has a certain correlation with the radiomic features representing tumor heterogeneity.

However, there were several limitations in our study. Firstly, the distribution of FDG in the body is also different in different physiological periods, which may affect the quality of the PET data to a certain extent. In the future, we will carry out stricter standardization on the data preprocessing. Secondly, the sample size of this study is relatively small and the source of cases is single. A large sample size and a multicenter test are needed for verification. In our future NPC studies, we plan to build models based on the combination of radiomic features and PET parameters as well as supplement external validation.

## Conclusion

The radiomic models based on ^18^F-FDG PET and MR images were valuable for the evaluation of the clinical stage of NPC. In the future, radiomics could become a more useful and economical tool for predicting the aggressiveness and distant metastasis of NPC. There was a correlation between the metabolic parameters and radiomic features, which reflects the correlation between the metabolic function and microstructure of tumor to some extent. In summary, the radiomic model based on ^18^F-FDG PET/MR has a high diagnostic performance in the evaluation of NPC staging, which is conducive to the accurate clinical staging of NPC after further verification.

## Data Availability Statement

The raw data supporting the conclusions of this article will be made available by the authors, without undue reservation.

## Ethics Statement

The studies involving human participants were reviewed and approved by the Ethics Committee of Zhejiang Provincial People's Hospital (NO. KT2018024). The patients/participants provided their written informed consent to participate in this study.

## Author Contributions

ZD designed and organized the research. LW, JN, and XG collected the clinical information and the imaging data. JL performed the ROI segmentation. QF, JL, and PP analyzed the data. QF contributed to the drafting and writing of the manuscript. All authors contributed to the article and approved the submitted version.

## Conflict of Interest

PP was employed by company GE Healthcare Life Sciences. The remaining authors declare that the research was conducted in the absence of any commercial or financial relationships that could be construed as a potential conflict of interest.
