# Supplementary material for: Radiomics Analysis and Correlation With Metabolic Parameters in Nasopharyngeal Carcinoma Based on PET/MR Imaging
Source: Front Oncol. 2020 Sep 8;10:1619. doi: 10.3389/fonc.2020.01619 (PMC7506153; doi:10.3389/fonc.2020.01619)
Supplement: Supplementary file 1 [file Data_Sheet_1.docx]

Supplementary Material

# Supplementary Data

1. The radscore formula for PET model:

"Radscore = 0.689*LongRunLowGreyLevelEmphasis_angle90_offset1_HLHL+1.377*LowGreyLevelRunEmphasis_angle90_offset1_LHHH+0.223*ShortRunLowGreyLevelEmphasis_AllDirection_offset1_HLLH+0.726*ShortRunLowGreyLevelEmphasis_angle0_offset1_LLLH+0.815*LowGreyLevelRunEmphasis_angle0_offset1_HLLL + 1.709"

2. The radscore formula for MR model:

"Radscore = -0.079*LowGreyLevelRunEmphasis_AllDirection_offset1_SD+-0.32*ShortRunHighGreyLevelEmphasis_AllDirection_offset4_SD+-0.093*GLCMEntropy_AllDirection_offset1_SD+-0.31*MinIntensity+-0.26*HighGreyLevelRunEmphasis_AllDirection_offset4_SD+-0.455*GLCMEntropy_angle0_offset4 + 1.063"
